# Determination of Iodate in Food, Environmental, and Biological Samples after Solid-Phase Extraction with Ni-Al-Zr Ternary Layered Double Hydroxide as a Nanosorbent

**DOI:** 10.1100/2012/145482

**Published:** 2012-04-24

**Authors:** Hossein Abdolmohammad-Zadeh, Keyvan Tavarid, Zeynab Talleb

**Affiliations:** Department of Chemistry, Faculty of Sciences, Azarbaijan University of Tarbiat Moallem, 35 Km Tabriz-Marageh Road, P.O. Box 53714-161, 5375171379 Tabriz, Iran

## Abstract

Nanostructured nickel-aluminum-zirconium ternary layered double hydroxide was successfully applied as a solid-phase extraction sorbent for the separation and pre-concentration of trace levels of iodate in food, environmental and biological samples. An indirect method was used for monitoring of the extracted iodate ions. The method is based on the reaction of the iodate with iodide in acidic solution to produce iodine, which can be spectrophotometrically monitored at 352 nm. The absorbance is directly proportional to the concentration of iodate in the sample. The effect of several parameters such as pH, sample flow rate, amount of nanosorbent, elution conditions, sample volume, and coexisting ions on the recovery was investigated. In the optimum experimental conditions, the limit of detection (3s) and enrichment factor were 0.12 **μ**g mL^−1^ and 20, respectively. The calibration graph using the preconcentration system was linear in the range of 0.2–2.8 **μ**g mL^−1^ with a correlation coefficient of 0.998. In order to validate the presented method, a certified reference material, NIST SRM 1549, was also analyzed.

## 1. Introduction

Since iodine was discovered as a new element in 1811 by the French chemist Barnard Courtois, the important roles of this significant nutrient substance have gradually been recognized. As an essential micronutrient in human growth and metabolism, iodine deficiency is a major public health problem for populations throughout the world, particularly for pregnant women and young children [1]. The most devastating outcomes of iodine deficiency are increased in brain damage, mental retardation, hypothyroidism, and cretinism [2]. Experts suggest that the simplest, most efficient means to against iodine deficiency are to add potassium iodate into salt. On the other hand, iodate is an important oxidant, which can oxidize many inorganic and organic compounds. This ion has been used for oxidation of metal ions, polyhydroxylated compounds, and catalytic applications at trace levels. In brief, iodate, due to its special functions as an essential micronutrient, marker of geochemically and biologically active processes, hazardous contaminant (in the form of radio-nuclides), and so on, plays an important biological and environmental role [3]. Therefore, the determination of this ion is required for better understanding processes for human health and environmental protection.

Several analytical techniques, such as spectrophotometry [4–11], spectrofluorometry [12, 13], chemiluminescence [14], colorimetry [15], ion chromatography [16–19], gas chromatography-mass spectrometry [20], transient isotachophoresis-capillary zone electrophoresis [21, 22], and electroanalytical methods [23–25], have been reported for the determination of iodate and only a few of them accompanied with a sample preparation step [26, 27]. However, as we know, the sample preparation processes have a direct impact on accuracy, precision, and quantification limits and are often the rate-determining step for many analytical methods. Therefore, analytical chemists continue to search for sample preparation procedures that are faster, easier, safer, and less expensive to perform yet provide accurate and precise data with reasonable quantification limits. Solid-phase extraction (SPE) techniques have been developed to replace many traditional liquid-liquid extraction methods. SPE methods utilize solid sorbents packed into disposable plastic or glass cartridges or embedded into Teflon or glass fiber disks. SPE techniques provide a means to process samples quickly, consume much less solvent, isolate analytes from large volumes of water with minimal or no evaporation losses, combine with different detection techniques, and can provide more reproducible results [28]. In order to control the analytical parameters such as selectivity, removal affinity, and adsorption capacity, the choice of suitable sorbents for SPE is very important.

Layered double hydroxides (LDHs), resembling the naturally occurring hydrotalcite, constitute an important class of lamellar ionic compounds and are represented by general formula [M_1−*x*_^2+^M_*x*_^3+^(OH)_2_]^*x*+^[A_*x*/*n*_^*n*−^ · mH_2_O]^*x*−^, where M^2+^ and M^3+^ are bi- and trivalent metal cations, *x* is equal to the ratio M^3+^/(M^2+^+M^3+^), and *A* is an interlamellar anion with charge *n* ^−^. The highly tunable LDH interlayer composition coupled with wide possible choice of anionic moieties and/or variation of composition of the host layers affords a large variety of solids with versatile catalytic and ion exchange/intercalation properties [29, 30]. Due to their permanent layer positive charge, high anionexchange capacity, large surface area, good thermal stability, water-resistant structure, and rapid regeneration, LDHs are considered as a new class of green nanosorbents for SPE of organic and inorganic anions [31, 32].

In this work, we have utilized the Ni-Al-Zr (NO_3_^−^) ternary layered double hydroxide, a newly synthesized LDH by our research group, as a nanosorbent for separation and preconcentration of iodate and its indirect determination as triiodide ions by spectrophotometric method. The effects of several parameters on the recovery of iodate ions were systematically investigated, and the proposed method was successfully applied to the determination of iodate ions in different food, environmental, and biological samples.

## 2. Experimental

### 2.1. Apparatus and Instruments

A T80 UV-Vis spectrophotometer (PG Instrument Ltd, England) with a wavelength range of 190–900 nm was used for recording the spectra and measuring the absorbance. The instrument equipped with dual source lamp (tungsten and deuterium for Vis and UV range, resp.), 1.0 cm quartz cell, and PMT detector. The spectral bandwidth and response time were set at 2 nm and 0.2 s, respectively. A 2 mL polypropylene cartridge (30 mm × 7 mm i.d.) (Shafa Co., Iran) packed with 0.2 g of Ni-Al-Zr (NO_3_^−^) LDH and fitted with small cotton beads at both ends to prevent material losses was used to preconcentrate the analytes in SPE procedures. The flow rate of solution through the column was controlled with an air-driven fluid pump model P34112 (Taiwan).

In order to structural study of the LDH, XRD measurements were performed on a Bruker AXS (D8 Advance) X-ray powder diffractometer (Cu K*α* radiation source, *λ* = 0.154056 nm) between 5 and 70° generated at 40 kV and 35 mA at room temperature. Samples for XRD were ground into powder and then pressed flat in the sample slot. In addition, FT-IR spectra (4000–400 cm^−1^) were recorded on a Vector 22 (Bruker, Germany) Fourier transform infrared spectrometer using the KBr disk method with a ratio sample/KBr of 1 : 100 by mass. A scanning electron microscope (SEM), model P Scan Vega 2 (Czech Republic), was additionally used to examine the morphological characteristics of the sorbent. Electrical furnace model Ex. 1200-4L (Exciton Co., Iran) and N_2_ gas (99.9995%, Azaroxide Co., Iran) were used in LDH preparation process. The pH values were measured with a Metrohm pH meter (model 827), supplied with a glass-combined electrode. An electronic analytical balance (Mettler Toledo, PB303, Switzerland) was used for weighting the solid materials.

### 2.2. Standard Solutions and Reagents

All chemicals used were of analytical-reagent grade, and all solutions were prepared with high-purity deionized water (Shahid Ghazi Co., Tabriz, Iran). Stock solutions of potassium iodate (1000 mg L^−1^) and potassium iodide (0.1 mol L^−1^) were prepared in deionised water, and the solutions of lower concentrations were prepared by appropriately diluting the stock solutions with deionised water. All the solutions of potassium iodide were kept in amber color glassware throughout the experiment. All salts used for the interference study, NaOH, KI, KIO_3_, HCl (37%), and LDH precursors, that is, purified nickel nitrate hexahydrate [Ni(NO_3_)_2_ · 6H_2_O, 99%], aluminum nitrate nonahydrate [Al(NO_3_)_3_ · 9H_2_O, 99%], and zirconium oxynitrate monohydrate [ZrO(NO_3_)_2_ · H_2_O, 99%], were purchased from Merck (Darmstadt, Germany). Suprapure HNO_3_ (65%) and H_2_SO_4_ (95–98%) were used for sample digestion. The pipettes and vessels used for the trace analysis were kept in 15% (v/v) nitric acid at least overnight and subsequently washed three times with deionized water prior to use.

### 2.3. Preparation of Nickel-Aluminum-Zirconium Ternary Layered Double Hydroxide

The Ni-Al-Zr (NO_3_^−^) LDH was prepared using a coprecipitation method. The synthesis was carried out under a N_2_ atmosphere, and all the solutions were prepared using deionized water to avoid contamination. In this work, the Ni^2+^ : (Al^3+^ + Zr^4+^) and Al^3+^ : Zr^4+^ molar ratios chosen for the synthesis of the LDH precursors were 3 : 1 and 0.7 : 0.3, respectively. For this purpose, 1.32 g Ni(NO_3_)_2_ · 6H_2_O, 0.38 g Al(NO_3_)_3_ · 9H_2_O, and 0.11 g ZrO(NO_3_)_2_ · H_2_O were added into 50.0 mL deionized water under vigorous stirring at room temperature. Then, 25 mL basic solution containing 0.5 mol L^−1^ NaOH was added dropwise to the salt solution, and pH of the solution was maintained at 8 under continuous stirring at room temperature. After aging, the Ni-Al-Zr (NO_3_^−^) LDH precursor suspension was transferred into a Teflon-lined autoclave and heated in an electrical furnace at 110°C for about 72 h. Afterward, the resulting precipitate was separated by centrifugation at 4000 rpm for 10 min, washed three times with deionized water, and dried at 60°C for 12 h.

### 2.4. Column Preparation

The solid-phase extraction column was prepared by introducing 200 mg of Ni-Al-Zr (NO_3_^−^) LDH into an empty 2 mL polypropylene cartridge using the dry packing method. Both ends of the column were plugged with a small portion of cotton to retain the nanosorbent in the column. Before loading the sample, the column was cleaned with 1.5 mL of 2 mol L^−1^ NaOH solution and conditioned by passing only 5 mL of deionized water through the column prior to each use.

### 2.5. Sample Preparation

#### 2.5.1. Rock Salt and Table Salt

500 mg of table or rock salt sample was transferred into a 50 mL volumetric flask, and after dissolving in deionized water, the solution was diluted to the mark with high-purity deionized water. In order to extract any insoluble particles in rock salt sample, the solution was filtered through the Rund filter paper (Blue band, no. 300210) prior to sample loading.

#### 2.5.2. Sea Water and Urine

After sampling, seawater was filtered through Rund filter paper to remove suspended particulate matter, and aliquots of 50.0 mL from sample solution were analyzed by following the procedure described in Section 2.6. In the case of urine sample, aliquots of 5.0 mL from this sample were transferred into a 50 mL volumetric flask and diluted to the mark with high-purity deionized water prior to sample loading.

#### 2.5.3. Milk Powder

Accurately measured amounts of powdered milk (Humana, 250 mg) and NIST SRM 1549 (nonfat milk powder) were separately digested with 10 mL H_2_SO_4_ (95–98%) and 5 mL HNO_3_ (65%) on a hot plate at a 250°C in the glass beaker to dryness [33]. After cooling the residue to room temperature and dilution with deionized water, the pH was adjusted to 5.5 by adding diluted NaOH solution. Then, the solution was transferred into a 50 mL volumetric flask and made up with deionized water. Finally, the sample was analyzed according to the following procedure.

### 2.6. General Procedure

For solid-phase extraction and preconcentration of iodate ions, aliquots of 50.0 mL of aqueous standard or sample solution containing iodate ions in the range of 0.2–2.8 *μ*g mL^−1^ (pH 5.5) were passed through the Ni-Al-Zr (NO_3_^−^) LDH nanosorbent in a column at a flow rate of 3.0 mL min^−1^. After loading, the retained analyte on the column was eluted with 1.5 mL of 2 mol L^−1^ NaOH solution at an elution rate of 1.0 mL min^−1^. As iodate could not be spectrophotometrically monitored, the extracted iodate ions were reduced to triiodide ions by adding 0.5 mL of 6.0 mol L^−1^ HCl and 0.5 mL of 0.1 mol L^−1^ KI as a reductant. The concentration of the obtained triiodide ions was monitored spectrophotometrically by measuring the absorbance of the solution at 352 nm, which corresponds to the iodate concentration. Thus, a one-step spectrophotometric method was applied to the iodate determination.

## 3. Results and Discussion

### 3.1. Characterization of Ni-Al-Zr (NO_3_^−^) LDH

**Figure 1:**
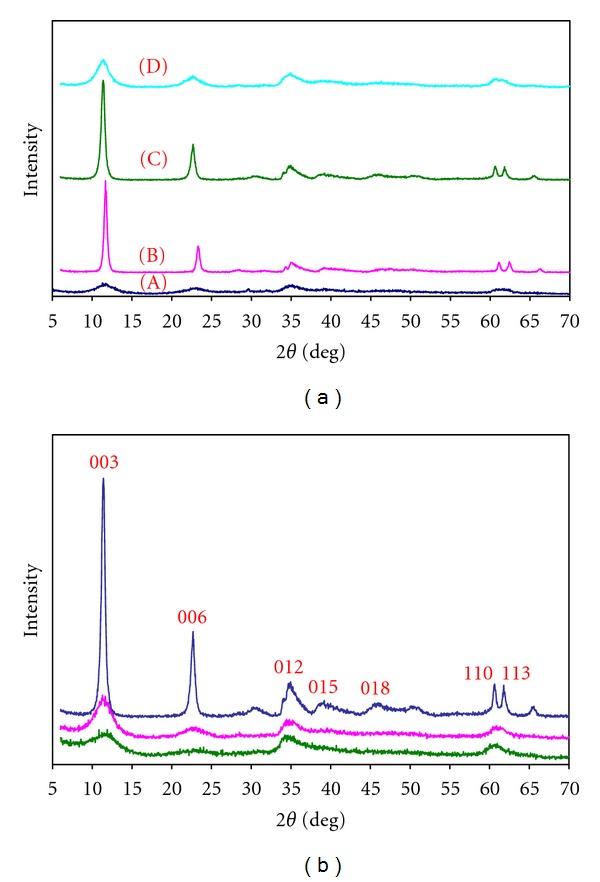
XRD patterns of Ni-Al-Zr (NO_3_^−^) LDH with different Ni/(Al+Zr) and Al/Zr molar ratios. (a) Ni/(Al+Zr) molar ratios of (A) 1 : 1, (B) 2 : 1, (C) 3 : 1, and (D) 4 : 1. (b) Ni/(Al+Zr) molar ratio of 3 : 1 and different Al/Zr molar ratios of 0.3 : 0.7, 0.5 : 0.5, and 0.7 : 0.3 from bottom to top, respectively.

The charge density and the anion exchange capacity of the LDHs may be controlled by varying the M^2+^/M^3+^ ratio. This ratio is important and could affect the retention efficiency of the analyte. Therefore, several Ni-Al-Zr (NO_3_^−^) LDHs were successfully synthesized with different Ni/(Al+Zr) molar ratios of 1–4 and Al/Zr molar ratios of 0.3 : 0.7, 0.5 : 0.5, and 0.7 : 0.3 by conventional coprecipitation method. A set of characterization was performed in order to get better insight into the structural properties of the obtained material, which has been used in this study as a solid-phase extractor.  Figure 1 shows the X-ray diffraction (XRD) patterns of as-synthesized dried hydrotalcite samples with different Ni/(Al+Zr) and Al/Zr molar ratios. They show sharp and intense reflections at around 2*θ* 11°, 23°, 60°, and 62° attributed to (0 0 3), (0 0 6), (1 1 0), and (1 1 3) planes characteristic of hydrotalcite-like materials with hexagonal crystal system. The positions of the remaining peaks are in agreement with the results reported by other researchers [34–36]. In Figure 1(a), the XRD patterns of Ni-Al-Zr (NO_3_^−^) LDH with Ni/(Al+Zr) molar ratios of 1 : 1, 2 : 1, 3 : 1, and 4 : 1 are shown from *a* to *d*, respectively. As can be seen, a good crystallinity was achieved in the Ni/(Al+Zr) molar ratio of 2 : 1 and 3 : 1 because these XRD patterns show narrow, symmetric, and strong lines at low 2*θ* values. In addition, the peaks with miller indices of (1 1 0) and (1 1 3) around 2*θ* 60° and 62° are also characteristic of well-crystallized LDH structure [37].  Figure 1(b) shows the XRD patterns of Ni-Al-Zr (NO_3_^−^) LDH with Ni/(Al+Zr) molar ratio of 3 : 1 and different Al/Zr molar ratios of 0.3 : 0.7, 0.5 : 0.5, and 0.7 : 0.3 from down to up, respectively. It was found that the thickness of the interlayer increases with an increase in Ni/(Al+Zr) molar ratio due to decrease in positive charge and electrostatic interaction between brucite-like mixed metal hydroxide layer and the anions in the interlayer. Therefore, in order to achieve high sorption capacity, the Ni-Al-Zr (NO_3_^−^) LDH with Ni/(Al+Zr) and Al/Zr molar ratios of 3 : 1 and 2.33 (0.7 : 0.3), respectively, has been used in this study as a nanosorbent for SPE of iodate ions.

**Figure 2:**
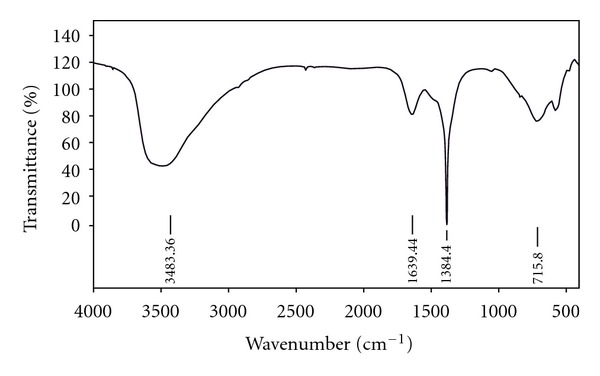
FT-IR spectrum of Ni-Al-Zr (NO_3_^−^) LDH.

Figure 2 shows the FT-IR spectrum of Ni-Al-Zr (NO_3_^−^) LDH. The absorption band around 3483 cm^−1^ is attributed to O–H stretching mode, caused by hydroxyl groups in the brucite-like layers and the interlayer water molecules. An absorption band at 1639 cm^−1^ is ascribed to water bending vibrations of the interlayer and/or adsorbed water molecules. The band with maximum peak at 1384 cm^−1^ can be assigned to stretching vibration of NO_3_^−^ ions intercalated in the interlayer gallery. The other bands observed in the low-frequency 400–1000 cm^−1^ region of the FT-IR spectrum are interpreted as the vibration modes attributed to metal-oxygen (M–O) and metal-hydroxyl (M–OH) groups in the lattice of LDH.

**Figure 3:**
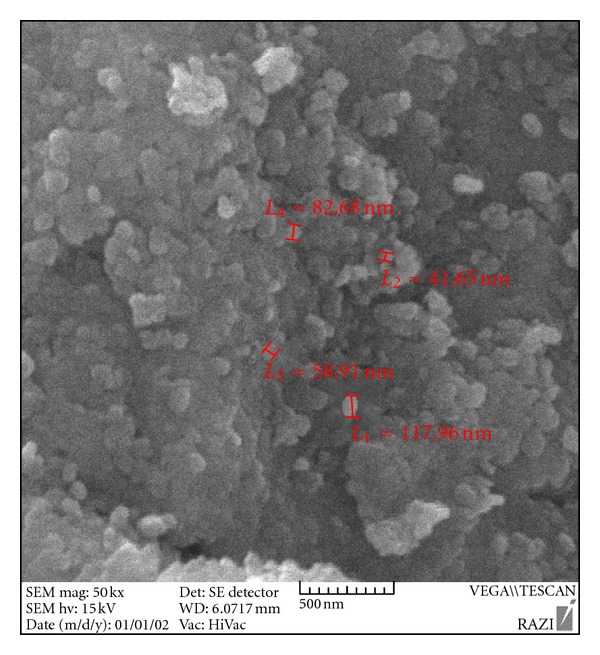
SEM image of Ni-Al-Zr (NO_3_^−^) LDH.

Scanning electron microscopy (SEM) was employed to explore the morphology of the nanosorbent. SEM image of the synthesized LDH, shown in Figure 3, reveals that Ni-Al-Zr (NO_3_^−^) LDH shows an aggregate that consists of crystallites was collected as small pseudospherical particles with approximate sizes in the range of 30–150 nm and stacking with each other, which makes plate-like morphology.

### 3.2. Optimization of SPE Conditions

To obtain the most suitable data from the presented preconcentration system, the effect of different parameters such as amount of LDH, pH of sample solution, sample loading flow rate, type and concentration of eluent, sample volumes and eluent flow rate on the retention efficiency has been studied and optimized. The optimization procedure was carried out by varying a parameter while the others were kept constant. A 5 *μ*g mL^−1^ iodate solution was used for all the measurements, and three independent experiments were carried out for each optimized variable. The recovery percentage, which was calculated from the amount of iodate ion in the starting sample and the amount of iodate ion eluted from the column, was used as the analytical signal.

#### 3.2.1. Effect of pH

**Figure 4:**
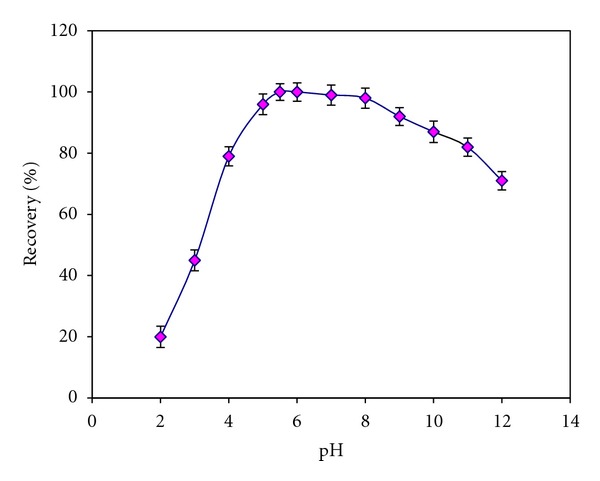
Effect of sample pH on the retention of iodate ions on Ni-Al-Zr (NO_3_^−^) LDH nanosorbent.

The influence of pH on the retention of iodate ions on Ni-Al-Zr (NO_3_^−^) LDH was investigated. The pH values of sample solution were adjusted in a range of 3.0–11.0 with minimum volume of 0.01 mol L^−1^ HCl and/or NaOH. As can be seen in Figure 4, the recovery of iodate ions depends on the pH of the sample solution and the optimum pH range was around 5–8. This result can be attributed to the increase of concentration of competing anions OH^−^ at higher pH (>8.0). However, at pH < 4, the uptake capacity was low due to dissolution of the layered materials in strong acidic media. Therefore, to achieve high efficiency and good selectivity, pH 5.5 was selected for subsequent works.

#### 3.2.2. Effect of Sample Loading Flow Rate

The influence of iodate ions retention on nanometer-sized Ni-Al-Zr (NO_3_^−^) LDH was investigated by varying the flow rate of the sample solution in the range of 1–5 mL min^−1^ and passing the 100 mL of iodate solution through the column using an air-driven fluid pump. The results showed that the flow rate variation in the range of 1–3 mL min^−1^ did not have a significant effect on the sorption of the iodate. It was found that the adsorption of iodate ion on Ni-Al-Zr (NO_3_^−^) LDH is relatively rapid. In order to achieve high frequency of analysis, a flow rate of 3 mL min^−1^ was therefore chosen for further studies.

#### 3.2.3. Optimization of Elution Conditions

**Figure 5:**
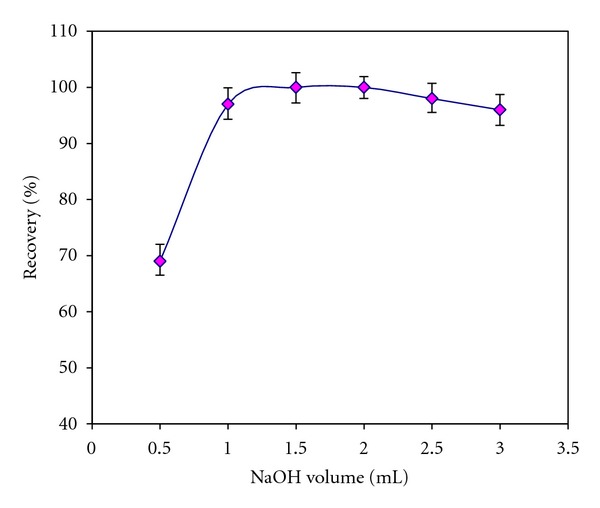
Effect of eluent volume on the recovery of iodate ions from Ni-Al-Zr (NO_3_^−^) LDH nanosorbent.

The nature of the eluent is of prime importance and should optimally meet three criteria: efficiency, selectivity, and compatibility. In addition, it may be desirable to recover the analytes in a small volume of solvent to ensure a significant enrichment factor. In this work, stripping of the retained analyte iodate ions from column was examined using various elution solutions such as NaOH, NaCl, and Na_2_CO_3_. The results showed that the best recovery was achieved when NaOH was used as eluent. The concentration, volume, and flow rate of the NaOH solution were also optimized. For this purpose, various concentrations (1.0–5.0 mol L^−1^) of NaOH were studied for the elution. Based on the obtained results, 2.0 mol L^−1^ NaOH was sufficient for complete elution of the adsorbed iodate on a Ni-Al-Zr (NO_3_^−^) LDH nanosorbent. By keeping the eluent concentration of 2.0 mol L^−1^ NaOH, the effect of elution volume (0.5–3.0 mL) on the recovery was also investigated. The percent of recovery of iodate ion increased by increasing the volume of NaOH up to 1.5 mL and remained constant afterward (Figure 5). Therefore, optimum volume of the eluent and its flow rate were chosen as 1.5 mL and 1.0 mL min^−1^, respectively.

#### 3.2.4. Effect of the Amount of Ni-Al-Zr (NO_3_^−^) LDH Nanosorbent

The effect of the amount of Ni-Al-Zr (NO_3_^−^) LDH nanosorbent on the retention of iodate ions at pH 5.5 was examined in the range of 50–300 mg. The results demonstrated that the quantitative recoveries (>95%) of the working ion were observed when the LDH used above 150 mg. Therefore, in the presented procedure, 200 mg of Ni-Al-Zr (NO_3_^−^) LDH is recommended.

#### 3.2.5. Sample Volume and Preconcentration Factor

An important parameter to control solid-phase extraction of real samples is the sample volume, which means the maximum sample volume that should be percolated through a given mass of sorbent after which elution of the analytes from the sorbent results in quantitative recoveries and high enrichment factor. For this aim, the volumes of sample solution containing 0.5 *μ*g mL^−1^IO_3_^−^ were diluted to 25.0, 50.0, 100.0, 150.0, and 200.0 mL. Then, retention and stripping processes were performed under the optimum conditions as described in previous sections. The recovery of iodate ions was found to be quantitative when sample volume was chosen between the ranges of 25.0–50.0 mL. Above 50.0 mL, the recovery decreased for the analyte. So, by analyzing 2.5 mL of the final solution after the preconcentration of 50.0 mL of sample solution, an enrichment factor was found as 20.

#### 3.2.6. Sorption Capacity

The capacity of the sorbent is an important factor that determines how much sorbent is required to quantitatively remove a specific amount of analyte from the solution. In this work, the sorption capacity of the LDH nanosorbent was calculated by the batch technique. For this process, 100 mg of the sorbent was added to 50.0 mL of solution containing 50 mg L^−1^ of iodate ions, stirred for 60 min with magnetic stirrer, and filtered through a filter paper. Enriched iodate ions onto LDH nano-particles were stripped with 2.5 mL of 2.0 mol L^−1^ NaOH prior to the determination. As a result, capacity of the Ni-Al-Zr (NO_3_^−^) LDH for iodate ions was found to be 7.7 mg g^−1^.

#### 3.2.7. Reusability of the Ni-Al-Zr (NO_3_^−^) LDH Nanosorbent

The potential regeneration and stability of the column were also investigated. The column could be reused after regenerating with 2.0 mL of 2.0 mol L^−1^ NaOH and 5.0 mL deionized water, respectively. Moreover, based on the obtained results, the Ni-Al-Zr (NO_3_^−^) LDH nanosorbent is stable as well as no carryover of analyte during SPE procedure, showing good reusability.

#### 3.2.8. Optimization of Detection Condition

In this study, an indirect method has been developed for the determination of iodate ions. Iodate reacts with iodide in acidic media to produce triiodide according to the following reaction:

(1) $$\begin{array}{cc} & {{\text{IO}}_{3}}^{-}\left(\text{aq}\right)+8{\text{I}}^{-}\left(\text{taken}\;\text{in}\;\text{excess}\right)+6{\text{H}}^{+}\left(\text{aq}\right)\\ & \;\;\to 3{{\text{I}}_{3}}^{-}\left(\text{aq}\right)+3{\text{H}}_{2}\text{O}\left(\text{aq}\right). \end{array}$$

The iodate ions could be monitored spectrophotometrically by measuring the absorbance of the solution at 352 nm, which is proportional to the triiodide concentration. According to the reaction, it is obvious that the reliability and sensitivity of the presented system depend on the complete transformation of iodate ions to triiodide. As the iodide concentration taken in excess (0.02 mol L^−1^), the significance parameter to perfect reduction of the iodate is the acid concentration. Our studies showed that adjusting pH of the 1.5 mL alkaline solution, containing the extracted iodate ions, at 2.5 by adding 0.5 mL of 6 mol L^−1^ HCl is a way to achieve this goal.

#### 3.2.9. Study of Interferences

In order to demonstrate the selectivity of the developed extraction method for the determination of iodate, the effect of alkali and alkaline earth metals and several oxyanions on the preconcentration and determination of the iodate ions was investigated. An ion was considered to interfere when its presence produced a variation of more than ±5% in the analytical signal of the sample. The results are shown in Table 1. As shown later, these results permit the application of Ni-Al-Zr (NO_3_^−^) LDH as an efficient nanosorbent for the interference-free solid-phase extraction and determination of iodate in different samples.

#### 3.2.10. Analytical Figures of Merit

Optimized experimental parameters and analytical characteristics of the method were given in Table 2. In the optimum conditions, a calibration graph was constructed for iodate by preconcentrating standard solutions according to the procedure under “experimental.” The linear concentration range was from 0.2 to 2.8 *μ*g mL^−1^ with a correlation coefficient of 0.998. The calibration function was *A *= 0.4184 *C *+ 0.0389, where *C* is the concentration of iodate ions in *μ*g mL^−1^. The limit of detection (3 s) was found to be 0.12 *μ*g mL^−1^. As the amount of iodate in 50.0 mL of the solution was concentrated to 2.5 mL, a preconcentration factor of 20.0 was achieved in this method. In order to study the precision of the method, a series of six solutions containing 0.5 *μ*g mL^−1^ iodate were measured at the same day. The relative standard deviation (RSD) was 2.5%.

#### 3.2.11. Validity of the Method and Analysis of Real Samples

In order to test the validity of the developed method, it was first applied to the determination of iodate in a standard reference material, NIST SRM 1549 nonfat milk powder. For this purpose, 250 mg of the certified material was digested according to Section 2.5.3, and the obtained solution was then analyzed by following the procedure described in Section 2.6. The certified amount of iodate in this SRM is 3.38 ± 0.02 *μ*g g^−1^, and the obtained value for iodate by using the presented method was 3.29 ± 0.12 *μ*g g^−1^ (mean of five determinations ± standard deviation), which is in good agreement with the certified concentration. Statistical analysis of these results using Student's *t*-test showed that there was no significant difference between actual and found concentrations at 95% confidence level. The method was then used for the determination of iodate in several food, environmental, and biological samples. The recovery tests were also performed by spiking the samples with different amounts of iodate before any pretreatment.  Table 3 shows the obtained results. As can be seen, the recovery values of different real samples were between 94.0% and 100.0%, which confirm the accuracy of the presented method.

## 4. Conclusions

LDH materials exhibit the advantages of facile manipulation, low-cost, large surface area, good thermal stability, and environment friendliness. It was found that the nanometer-sized Ni-Al-Zr (NO_3_^−^) ternary layered double hydroxides with Ni/(Al+Zr) ratio of 3 : 1 and Al/Zr ratio 2.33 (0.7 : 0.3) is stable and has a great potential as a sorbent for the preconcentration of iodate ions. This methodology gives good accuracy, low detection limit, high uptake capacity, excellent precision, and relatively high kinetic sorption on the iodate ions, which show its efficiency in trace analysis in various samples with complicated matrix. Comparison of the presented method with other methods reported in the literature shows that the RSD and LOD of the method are comparable or better than other reported methods (Table 4). In conclusion, we encourage the use of Ni-Al-Zr (NO_3_^−^) LDH nanosorbent for the preconcentration and determination of iodate ions in routine analytical laboratories.

## Figures and Tables

**Table 1:** Tolerance limits of interfering ions in the determination of 0.2 *μ*g mL^−1^ iodate.

Ions	Interferent-to-analyte ratio
Ca^2+^, Al^3+^, Mg^2+^, Fe^3+^	1000 : 1
CO_3_^2−^, ClO_4_^−^, Cl^−^, CH_3_COO^−^, Br^−^	500 : 1
SO_4_^2−^, PO_4_^3−^, F^−^	100 : 1
V(V), Mo(VI), Cr(VI)	50 : 1
BrO_3_^−^	1 : 1

**Table 2:** Optimum conditions and analytical performance of the presented method for iodate determination.

Experimental conditions	Unit	
Working pH	—	5.5
Amount of Ni-Al-Zr LDH	(mg)	200
Sample volume	(mL)	50
Sample loading flow rate	(mL min^−1^)	3
Eluent concentration	(mol L^−1^)	2
Eluent volume	(mL)	1.5
Elution flow rate	(mL min^−1^)	1
Iodide concentration	(mol L^−1^)	0.1
Detection pH	—	2.5
Final volume	(mL)	2.5
Wavelength	(nm)	352

Analytical parameters	Unit	

Linear range	(*μ*g mL^−1^)	0.2–2.8
Intercept	—	0.0389
Slope	—	0.4184
Detection limit^a^	(*μ*g mL^−1^)	0.12
Correlation coefficient	—	0.998
Relative standard deviation (*n* = 6)^b^	(%)	2.5 (0.5)
Enrichment factor^c^	—	20

^a^Calculated as three times the standard deviation of the blank signal.

^b^Value in parentheses is the iodate concentration (*μ*g mL^−1^) for which the RSD was obtained.

^c^Enrichment factor calculated as the ratio between the volume of the initial aqueous solution and the final elution volume.

**Table 3:** Determination of iodate in different real samples (results of recoveries of spiked samples).

Samples	Added IO_3_^−^ (*μ*g g^−1^)	Found IO_3_^−^^a^ (*μ*g g^−1^)	Recovery (%)
Table salt (1)^b^	—	3.6 ± 0.8	—
	10.0	13.3 ± 0.8	97.0
Table salt (2)^c^	—	5.2 ± 0.9	—
	10.0	14.8 ± 0.6	96.0
Table salt (3)^d^	—	3.3 ± 0.5	—
	10.0	12.7 ± 0.8	94.0
Rock salt^e^	—	1.7 ± 0.3	—
	10.0	11.5 ± 0.2	98.0
Milk powder^f^	—	2.5 ± 0.4	—
	10.0	12.2 ± 0.7	97.0

Samples	Added IO_3_^−^ (*μ*g mL^−1^)	Found IO_3_^−^^a^ (*μ*g mL^−1^)	Recovery (%)

Seawater^g^	—	0.1 ± 0.4	—
	0.1	0.2 ± 0.6	100.0
Urine^h^	—	0.1 ± 0.1	—
	1.0	1.1 ± 0.1	100.0

^a^Mean of three experiments ± standard deviation.

^b^Obtained from Ehteram Co. table salt, Khoy, Iran.

^c^Obtained from Rahnema Co. table salt, Tabriz, Iran.

^d^Obtained from Ghohar Dane Co. table salt, Sanandaj, Iran.

^e^Mineral salt, Urmia, Iran.

^f^Obtained from the local pharmacy (Humana Co. milk powder).

^g^Collected from Caspian Sea, Nowshahr, Iran.

^h^Healthy human urine, 25 years old man.

**Table 4:** Comparison of the presented method with other reported methods for iodate determination.

Detection system	LOD (*μ*g mL^−1^)	Linear range (*μ*g mL^−1^)	RSD (%)	Reference
FI-UV-Vis	0.02	0.1−30	1.2	[4]
Reverse FI-UV-Vis	0.008	0.2−3	0.9	[8]
Sequential FI-UV-Vis	0.05	0.05−10	0.8	[9]
TICP-CZE-UV	0.0035	Up to 5	1.08	[21]
LPME-microvolume UV-Vis	0.001	0.0075–0.175	4.2	[27]
SPE-spectrophotometry	0.12	0.2–2.8	2.5	This work

Notes: LOD: limit of detection; RSD: relative standard deviation; LPME: liquid-phase microextraction; FI: flow injection; TICP-CZE-UV: transient isotachophoresis-capillary zone electrophoresis with UV detection.
